# Some pupils should know better (because there is better knowledge than opinion). Interim findings from an empirical study of pupils’ and teachers’ understandings of knowledge and big questions in Religious Education

**DOI:** 10.1007/s40839-021-00155-5

**Published:** 2021-10-22

**Authors:** Alexis Stones, Jo Fraser-Pearce

**Affiliations:** UCL Institute of Education, London, UK

**Keywords:** Religious Education, Epistemic injustice, Knowledge, Big questions, Opinion, Epistemic literacy

## Abstract

In this paper, we draw on interim findings of our research project on Religious Education (RE), knowledge and big questions. We have found Miranda Fricker’s concept of epistemic injustice useful in our analysis—that is, the notion that a person can be wronged “specifically in their capacity as a knower (Fricker 2007, 1). In interviews with Key Stage 3 pupils (aged 12–14) we found that for many pupils, their capacity to know was hindered by the prioritisation of respect for opinion. Where opinion is considered something not to be questioned, this seems to be a key indicator of epistemic disadvantage while some pupils valued and could employ criticality when considering knowledge claims (including opinions). Epistemic advantage in this way exacerbates epistemic injustice, broadening a gap between the epistemic haves and have-nots. This research is part of a larger project where we attempt to answer the question: ‘Does Religious Education have a distinctive contribution to make to the development of epistemic literacy?’. We begin with our account of epistemic literacy underpinned by Young’s powerful knowledge (Young and Muller 2010) and contextualise our data with discourses about knowledge and school education. We focus largely on the emergent theme of (respect for) opinions and we argue that the prioritisation of respect in RE is (for some pupils) a barrier to knowledge. We go on to explore why this matters for individuals, society and RE.

## Introduction

Our assertion that some pupils should know better can also be read as an assertion that, as a Religious Education (RE) subject community, we should enable them to do so. This rests on more than an assumption that we can. In this paper, we draw on the interim findings of our research project on RE, knowledge and big questions. At the time of writing, our analysis of our data is in progress. In conducting this analysis, we have found Miranda Fricker’s concept of epistemic injustice useful—that is, the notion that a person can be wronged “specifically in their capacity as a knower” (Fricker, 2007, p. 1). In interviews with Key Stage 3 pupils (aged 12–14) we found that for many pupils their capacity to know was hindered by the prioritisation of respect for opinion. Indeed, considering opinion as something like sacrosanct and not to be questioned seems to be a key indicator of epistemic disadvantage. Not all pupils are so disadvantaged. Some of the pupils we spoke to valued and could employ criticality when considering knowledge claims (including opinions). That some pupils are epistemically advantaged in this way exacerbates epistemic injustice, broadening a gap between the epistemic haves and have-nots.

It is because some pupils understand knowledge and knowledge claims in sophisticated and critical ways that we know that we can enable other pupils to “know better”. We think this is important for individuals and society. The importance for the latter goes beyond matters of equity and justice. Some of the dangers surrounding a credulous populace are articulated in the introductory quote from Clifford and also here by Susan Haack:

[T]he more people are easily duped, the more likely it is that charismatic but crazy politicians will gain power, the more briskly well-advertised but ineffective or even dangerous ‘cures’ will sell, and the greater the chances are that juries will convict, or acquit, on other grounds than the strength of the evidence...

(Haack, 2015)

The research presented in this paper is part of a larger project, in progress at the time of writing. In this larger project we are attempting to answer the question: ‘Does Religious Education have a distinctive contribution to make to the development of epistemic literacy?’. We begin this paper with our account of epistemic literacy which is underpinned by Young’s powerful knowledge (Young & Muller, 2010). In advance of outlining our research design, we contextualise our data with a presentation of salient popular discourses about knowledge and school education. In the presentation and discussion of data we focus largely on the emergent theme of (respect for) opinions, as this was found in the majority of pupil interviews. Finally, we argue that the prioritisation of respect in RE is (for at least some pupils) a barrier to knowledge. We elaborate on why this matters for individuals and society.

### What do we mean by ‘epistemic literacy’?

By ‘epistemic’ we mean in relation to knowledge(s) and knowing. By ‘literacy’ we refer to the ability to do something with that knowledge. Regarding the context of RE, we appreciate the influence of other literacies and epistemic frameworks in developing our account of epistemic literacy; including religious literacy (Hannam et al., 2020), religion and worldviews literacy (Shaw, 2020), epistemic switching (Gottlieb & Wineburg, 2012) and epistemic insight (Billingsley et al., 2013). Recent theoretical formulations of religious literacy stress the rejection of imperialist understandings of literacy in which the powerful (in a) community decide what is the dominant language (Hannam et al. 2020). Religion and worldviews literacy, meanwhile, calls for one’s reflexivity combined with the appropriate disposition and tact that results from (1) knowledge of the actual religious/non-religious landscape, and (2) an understanding of the category of religion/worldview (Shaw, 2020). We build on these literacy projects to highlight the importance of acknowledging different kinds of knowledge, and ways of knowing, while recognising the variety of engagements with knowledge. These literacies chime with a ‘capabilities approach’ to education (Nussbaum, 1988; Sen, 1999) in understanding one’s literacy as an ongoing project, rather than a finite level/standard to be reached.

Epistemic switching and insight are both grounded in empirical research and equally pertinent for us. Epistemic switching was identified when participants (religious leaders and academics) in a study were found to unconsciously ‘switch’ between epistemic positions (academic, nationalist and religious), depending on context and knowledge claim (Gottlieb & Wineburg, 2012). Epistemic insight (Billingsley et al., 2013) was developed as an intervention in response to pupils’ lack of insight into the distinct and different knowledge structures found in RE and Science due to assumptions that they are in conflict. In both cases, ‘epistemic’ usefully describes ways of knowing and accounts of what constitutes reliable knowledge.

### Powerful knowledge, capabilities and social justice

Despite popular usage and interpretations of ‘powerful knowledge’ across contemporary educational policy (see below), our proposal for epistemic literacy is underpinned by Michael Young’s powerful knowledge as a social justice project grounded in his perspective as a sociologist of education (see Young, 2008). As a sociologist Young reminds us that knowledge is not merely a matter for education but for society as a whole. This has significant implications for an RE concerned with epistemic literacy.

Young’s project is an answer to an ongoing and underlying question about the purpose of schools. He responds to this question with the identification of the kind of knowledge that has power to take pupils beyond their everyday experiences. The ‘big questions’ concerning religion and science are not confined to RE and exist in the public and private domain as well—that is, the questions and our answers to them exist in everyday experience. Although a staple feature of the RE curriculum, big questions are prone to pupils’ engagement with knowledge(s) that remain in the epistemic environment of their everyday knowledge and experiences. Such knowledge and experiences are certainly germane where big questions are concerned but they are insufficient for epistemic literacy.

Building on Young, we argue elsewhere (Stones and Fraser-Pearce, 2021) that epistemic literacy can only be developed if pupils’ (and teachers’) relationships with knowledge are taken more seriously than seems to be the case with popular accounts ‘knowledge-rich’ curricula. Young’s attention to relationship with knowledge is influenced by sociologist, Bernard Charlot, who conceives schools as “places where the world is treated as an ‘object of thought’ and not as a ‘place of experience’” (Young, 2015, p. 98). By this, he refers to the distinction that he advocates should be made between the generalisations one can make about a disciplinary concept and one’s experience in relation to the concept in question. Grasping the relationship(s) between these two categories (crudely conceived as objective and subjective) with awareness of one’s own partiality, necessary tact and criticality, would be to demonstrate epistemic literacy.

But this is no mean feat. Big questions and their responses, more often than not, emerge from everyday experiences. We are concerned to facilitate a relationship with knowledge that acknowledges personal experience (subjective knowledge) and recognises it as such, *and* recognises the value of disciplinary (objective) knowledge. Neither knowledge nor our relationship to it is static—over time new disciplinary concepts are encountered and new experiences gained. For this reason, we have drawn on the ‘capabilities approach’ to education (Lambert et al., 2015; Nussbaum, 1988; Sen, 1999). Capabilities are pertinent for our view of epistemic literacy as they include epistemic capabilities, freedom and agency in the present *and* future lives of learners. Epistemic literacy refers to the navigation of knowledge and the process of knowing both during and beyond school life, as well as acknowledging the situatedness of navigating knowledge, and knowing, within and outside school.

Rather than focus primarily on economic and physical limitations, Sen’s capabilities approach offers a theory of justice which emphasises the importance of an individual's *sense* of her capability that defines her freedom and limitations (Sen, 1999). Sen highlights the importance of education in unlocking freedom to cultivate the agency that can unlock further freedoms. We posit that enabling epistemic literacy, which constitutes a capabilities approach, can challenge epistemic injustice (Fricker, 2007), and therefore hinder the development of a credulous populace (Clifford, 1877 and Haack, 2015). Following Sen’s ‘unlocking’ process, epistemic literacy seeks to “show the strings”, or mechanisms, of knowledge formation in a way that reveals the nuances and varieties of knowledge to enable the epistemic freedoms and agency of the knower.

Powerful knowledge shares comparable and compatible aspirations with capabilities, seeking to distribute and develop knowledge beyond the stronghold of the powerful (Young, 2008). Young’s conceptualisation of knowledge builds on a Durkheimian (Durkheim, 2001) view of knowledge in communities where the religious leader mediated the sacred knowledge from its divine source and interpreted it for the lay people. Although originally developed through study of aboriginal societies, this ‘downward’ movement of knowledge from the sacred to the profane is found in classrooms when it is the teacher who ‘has’ the knowledge and transforms or adapts it for pupils through pedagogical approaches. Young also acknowledges the contrasting direction of a Vygotskyan approach to knowledge (Vygotsky, 1987), in which the educator values the pupil’s experience and builds on their everyday knowledge to bring them to an understanding of the relevant specialist knowledge. Young’s claim is that schools exist to enact a social justice in which expertise are made available to all (not just the powerful), enabling epistemic access to powerful knowledge—a search for truth in its broadest sense and the potential to imagine the yet unimagined (Young, 2015).

Despite the socially just intentions of maintaining the authority of expert knowledge, rather than a ‘pupil-led’ curriculum that Young is so keen to dismiss as inferior, his framework presents some problems for RE. Where ‘expert’ knowledge relates to disciplines as the best form of knowledge available, everyday knowledge is considered something to move *beyond* (Young, 2015). RE curricula typically include (a) accounts of religious and non-religious experience, and (b) pupils’ personal engagement with universal concepts, such as death, war, hope and freedom. Powerful knowledge tacitly raises the status of the disciplinary accounts of experience and, by implication, reduces that of the pupil’s experience. Vernon (2020) highlights this problem and proposes the need for a multi-direction of travel of authority to allow for a continuum to emerge, in which different sites of inspiration are made available to pupils. Indeed, this would seem to cater for an epistemically plural classroom in which existential questions are met with diverse references, associations and experiences.

Given the significant influence of Critical RE (Wright, 2007) in the genealogy and culture of contemporary RE, we want to make it clear that our understanding of powerful knowledge is by no means incompatible with RE grounded in critical realism, despite the sociological nature of Young’s project. Critics might suggest a truth ‘out there’ bears no relevance to a truth that is constructed, according to a sociological epistemology. But this apparent problem is a symptom of the different frameworks to understand reality, and not a claim to reality itself. Although Critical RE acknowledges ontological realism, the social construction of truth is recognized through its tenets of the limits of human understanding of the truth (epistemic relativity) and the need for rational assessment. The truth exists, but all we have access to is our construction of it.

### Contemporary discourses on knowledge, school education and Religious Education

Reforms to the school curriculum led by education secretary, Michael Gove, under the Conservative-Liberal Democrat coalition government (2010–2015), started a public discourse about the role of knowledge in schools. Introduced as an antidote to the perceived lack of rigour in the English curriculum, Gove’s changes reflected the strong influence of American education author, E.D. Hirsch, who prizes the development of ‘cultural literacy’ through ‘core knowledge’ acquisition as a gap filling process to solve social and economic inequality (Hirsch, 1987). Learning a list of national rivers soon became a famous example of this new ‘knowledge turn’ which was (and remains) simultaneously unconvincing to critics and a welcome return to traditional educational values for supporters. Curriculum reforms resulted in a new specification for General Certificate of Secondary Education and Advanced Levels (public examinations for secondary school aged pupils) in which the ‘knowledge turn’ was enacted through a greater focus on substantive knowledge, and curriculum content (over skills) increased considerably as a result.

Minister for schools, Nick Gibb, continued the turn to a knowledge-rich education with the introduction of learning techniques offered by cognitive science and a focus on assessment. Knowledge was now conceived as filling the attainment gap and “a driver of true meritocracy” that Hirsch promised (Gibb, 2017, 2021). The prevalence of knowledge booklets and organisers for subjects in schools is a reminder that the knowledge-rich culture has entered the education lexicon and now travels beyond policy makers to reach the minds and homes of teachers, pupils and their families. This knowledge is perceived as core and vital to subjects, and yet is defined by exam board specifications or school frameworks for assessments.

A discussion of the public discourses surrounding knowledge is beyond the scope of this paper, but we can say something of the discourses that Covid-19 has brought to conversations around knowledge and authorities in relation to science. “Science says”, “Trust the science”, “Don’t trust science” are phrases heard and read in populist coverage of the pandemic. The discourse erroneously indicates a reductive notion that ‘science’ is a unified voice, a single body of knowledge or scientism. Climate change, vaccinations and AI similarly bring scientific perspectives and public opinion into a vibrant, diverse and populist discourse in which the authority of knowledge is debated in sensationalist terms, fuelling a ‘click bait’ economy in social media and rallying protest movements.

In their review of the literature on the ‘post truth condition’, Barzalai and Chinn (2020) highlight the epistemic crisis in four areas: “not knowing how to know, fallible ways of knowing, not caring about truth (enough), and disagreeing about how to know truth”, in which distrust, misinformation and rumours flourish today through social media in ways that are comparable to the Middle Ages (Barzalai and Chinn 2020, p. 107). Meanwhile, warnings of ‘fake news’ prevail in many populist narratives that point to the degradation of the epistemic environment resultant of an increasing absence of reliability and security in epistemic institutions (Blake-Turner, 2020). Related in some ways, conspiracy theories have a long-standing relationship with epistemic authority. They intensify in times of crisis, rely on emotion, identity, impact on decisions regarding one’s health and justify a disengagement from politics and knowledge institutions (Douglas and Sutton 2018). Although they range from a desire for unification, they are also seemingly contradictory (ibid.). Despite this range, there is unity in the acceptance of a grand conspiracy narrative which echoes the concept of the ‘explanatory space’ (Preston & Epley, 2009) that explains everything and requires minimum explanation. ‘Lazy thinking’ is less the culprit where implausible claims are accepted, with a recent and large-scale study pointing to a lack of analytical and evaluative skills as the enabler in holding these claims (Martire et al., 2020).

The claim that educators of RE want their pupils to think critically when considering knowledge and truth claims is perhaps taken for granted in the assumptions of a liberal education. Indeed, the ‘skills’ of reflection, evaluation and analysis have been embedded in a typical RE curriculum for decades and they define the subject in many ways. The canonical pedagogical scholarship of critical RE (Wright, 2007), conceptual enquiry (Erricker, 2010) and ethnographic or interpretive approaches (Jackson, 1997; Nesbit 2004) and, more recently, the notions of religious literacy (Hannam et al., 2020) and religion and worldviews literacy (Shaw, 2020), all highlight the crucial role that criticality plays in pupils’ relationships with knowledge and truth claims. Simultaneously, ‘community cohesion’, or ‘social harmony’, has a strong footing in the perceived purpose of the subject and the subject’s role in broader aims of school education. Conceived compatibly, criticality supports an informed community cohesion that depends on the tolerance of freedom of belief while “recognising and living alongside those whose beliefs are fundamentally incompatible with one’s own” (Wright 2007, p. 334). On the other hand, opposition criticises the epistemic impoverishment that results from ignoring difference and highlights instrumentalisation of the subject through this perceived purpose (see Hussain, 2018).

### Research design

In this paper we focus on one aspect of our current research project, in which we are attempting to answer:

- How far, if at all, do students and RE teachers understand, navigate and apply religion and science as discrete explanatory systems (Preston & Epley, 2009) in relation to big questions?
- What do students and teachers know about knowledge in relation to religion and science? How do they define knowledge and categorise ‘knowledges’?
- How far, if at all, do teachers and students use and engage with the multiple disciplines of RE in the development of their knowledge, understanding and responses to big questions?
- What are teachers’ and students’ views on the appropriateness and viability of epistemic literacy as an educational aim?

For the purpose of this paper, we are concerned with pupils’ and teachers’ perspectives in relation to big questions and knowledge.

In our project as a whole, we opted for a mixed-methods design comprising: semi-structured interviews with RE teachers and groups of Key Stage 3 pupils; observations of Key Stage 3 RE lessons; and an online teacher survey. In this paper, we draw upon data collected in teacher interviews and pupil group interviews. We visited eight schools around England and interviewed about three teachers, and five or six pupil groups, in each school—amounting to 20 teachers and 36 groups of pupils. All interviews were conducted by both authors of this paper. Our sampling was opportunistic as we drew upon our existing contacts with schools. We were concerned that our sample be diverse and were pleased that the range of participating schools included: rural, suburban and urban schools; boys, girls and co-educational schools; schools of religious character and those not of a religious character; independent, grammar and comprehensive schools; and schools from a range of English counties with the Northmost being in Yorkshire and the Southmost in Berkshire.

As is the nature of semi-structured interviews, they allowed us to pursue relevant lines of inquiry in relatively freely and in depth. As such, and again as is the nature with semi-structured interviews, there was some variation in the structure and flow of interviews and the phrasing of questions. Nevertheless, all interviews began by presenting participants with these examples of what we consider to be big questions:

- Why did the universe begin?
- Is there life after death?
- How do we know what being good or bad is?
- How do we know whether something is right or wrong?
- How do you know if something is true or false?

Participants were invited to offer further examples and asked “What makes a question a big question?” We asked a range of questions on big questions, including one to pupils on how important big questions are in their lives. We then turned to knowledge, including the following asked of pupils:

- What kind or kinds of knowledge would you need to answer big questions?
- How would you know you are using the right kind of knowledge? (Knowledge you could rely on/trust?)

Similarly, the following were included in the questions we asked teachers:

- What kind or kinds of knowledge would/do pupils need to answer big questions?
- How would they know if they are using the right kind of knowledge? (Knowledge they could rely on/trust?)

We did not present an understanding of ‘knowledge’ to participants (even when they asked), as we wanted to find out about participants’ understandings and interpretations.

Ethical approval was obtained for the project through the usual university process. Interviews were arranged by teachers at times convenient to participants and in places where interruptions were minimal. Voluntary informed consent was obtained by all participants, and parental/carer permission obtained for pupils. We took care to give all participants the opportunity to ask any questions immediately in advance of commencing interviews, and made it clear that they could withdraw from part or all of the interview at any time without consequence. As this project is in progress, data continues to be held (confidentially and anonymously).

### Findings and discussion: to respect is to refrain from questioning knowledge claims

#### Pupils’ and teachers’ perspectives on big questions

All individual teachers and pupil groups defined big questions in terms of their answers – that is, in terms of whether they can be answered, how they might be answered, and the kinds and numbers of answers they might have. At least initially, most of the participants who said big questions cannot be answered conflated the possibility of answering a question with the possibility of there being an answer. In some interviews, we explored the notion that there are no answers: did participants mean that there are no answers, or is it possible that there is an answer but it is not possible to know it? Some agreed the latter might be the case. Some participants said that answers to big questions matter; that they have an impact on our lives. Big questions were commonly understood by our participants as: having been around for a long time, questions that everyone has, taking a long time to answer and difficult. All of the teachers and most of the pupils said they find big questions interesting. A minority of pupils said they did not find them interesting and did not enjoy talking about them (although the pupils in one of the groups professing this engaged in the discussion enthusiastically). Most participants agreed that the questions matter even though they are really difficult (or even impossible) to answer. One pupil was of the view, in relation to big questions, that “if it doesn’t affect you, don’t worry about it”.

None of the participants took issue with the examples of big questions we offered, and participants expressed broad agreement over the nature of big questions. Participants suggested their own questions (these are listed later in this section) in response to being asked for examples of big questions other than those we had offered. Not all respondents expressed the questions in exactly the same words—for example, variations of ‘Is there a God?’, include ‘Does God exist?’ and ‘Is God real?’. Where we are sufficiently confident of a common meaning, and for ease and clarity of presentation, we have presented a single version of the question below. Where we are less confident of a common meaning, *or* where we think the variation is significant, we have retained distinct wording. To illustrate this, we have not included ‘Is an idol a god?’ as a variation of ‘What is God?’. An example of the latter is found in our distinction between ‘Did God create the universe?’ and ‘Did God create the universe or was it the big bang?’. The distinction is significant because the second variation only allows for two possible answers.

This reduction of options by the time pupils are 12–14 years old is reminiscent of what Ashley (2005) calls “early closure”. He offers an example from a 14-year-old research participant: “I don’t want to hear anything more about the environment because I learned everything I need to know at primary school” (Ashley, 2005, p. 190). Although this pupil refers to environmental education, the point is surely transferable to other subjects. We have seen that pupils and teachers understand big questions as both important and difficult. Given the gravity of these questions, “early closure [should be] prevented and [learning should continue] to the point at which the learner is able to marshal a wide range of arguments of increasing sophistication” (Ashley, 2005, p. 192). This would not negate pupils (or any of us) offering interim responses, but it would mean that the process of reaching a conclusion looked more like an “extended project” (Ashley, 2005, p. 192) than a sequence of lessons.

‘Did God create the universe or was it the big bang?’ was suggested in eight pupil group interviews whereas ‘Did God create the universe?’ was offered in one pupil group interview. Although the latter only states one option (that God created the universe), it does not limit other possible options and therefore does not indicate “early closure”. Neither does it indicate a conflict model of science and religion, or as Preston and Epley (2009) put it, a model in which science and religion compete for explanatory space. The former example does suggest such an understanding.

Big questions suggested by multiple participants:

- Is there a God? (Suggested in 20 of the 36 pupil groups and by eight out 20 teachers)
- What is the meaning and purpose of life?/Why am I here? (Eight pupil groups and three teachers)
- What is God? (Six pupil groups and two teachers)
- Did God create the universe or was it the big bang? (Eight pupil groups)
- If God is good, why do bad things happen? (Five pupil groups, one teacher)
- What does it mean to be human? (Two pupil groups, four teachers)
- How were humans brought into existence? (Five pupil groups)
- Who is the right God?/What is right the religion? (Four pupil groups)
- Do aliens exist? (Four pupil groups)
- How do we lead a good life? (Four teachers)
- Is this life/world we experience real? (Three pupil groups/one teacher)
- What does it mean to know?/How do we know? (Three teachers)
- Did the chicken or the egg come first? (Three pupil groups)

The questions teachers and pupils offer are not trivial even if some seem so at first. For example, one of the pupils who offered the chicken and egg conundrum used this as a springboard to raise questions about the beginning and development of animal life. Another pupil (in a different school) gave it as an example of an impossible big question and compared it to “what came before God?”.

### Pupils’ perspectives on using knowledge to answer big questions

Having noticed the serious nature of the big questions offered by pupils, as well as the fact that they consider big questions to be difficult and important, we were surprised that opinion was frequently cited by pupils as the main kind of knowledge needed to answer big questions. The word ‘opinion’ appears 743 times in the dataset. The majority of these appear in pupil group interviews, for example:

[Interviewer: How do you decide whether you’ve come up with the right answer to a big question?]

Pupil: “Your opinion.”

“Maths doesn’t have your opinion, it’s what’s right and wrong, the answer is right or wrong. But in RE there is no right or wrong, it’s your opinion.”

“Because everyone’s allowed to have like their own opinion. So, I guess the only real knowledge you need [to answer big questions] is your own opinion.”

Our review of literature had led us to anticipate particular kinds of responses – for example, we were anticipating that at least some participants would subscribe to a conflict model of science and religion where both compete for the same explanatory space (Preston & Epley, 2009). We were not prepared for the prevalence of opinion as (often times decisive) knowledge. We argue that individuals who prioritise opinion in answering big questions, and are reluctant to challenge opinions, are at an epistemic disadvantage. Where such an understanding of (or relationship with) knowledge has been intentionally nurtured, this constitutes epistemic injustice. In Fricker’s words: “a distinctively epistemic kind of injustice… wrong done to someone specifically in their capacity as a knower” (Fricker, 2007, p. 1).

One might suppose that these attitudes to opinion and knowledge simply constitute lazy thinking, but there is nothing in the data to suggest this. The data suggests that the prioritisation of opinion results in part at least from a well-intentioned but, we argue, miseducational (mis)understanding of the purpose of RE. In the words of one of the pupil participants:

RE is there to teach you to respect other religions and their beliefs.

For most of our pupil participants, to respect is to refrain from questioning knowledge claims. As evidenced in the following interview excerpt, this aversion to challenging opinion can extend to pupils comfortably describing a single claim to knowledge as at once being “right” *and* “wrong”:

Pupil one: “So to science your opinion [that the earth is flat] would be wrong, but no opinion is actually wrong.”

Pupil two: “... unless it is, like … an opinion about someone, like not a very nice opinion.”

The exception expressed by pupil two reinforces the primary concern with respect: opinions should be respected unless those opinions themselves are disrespectful. In such cases, the key criterion for assessing knowledge claims seems to be a moral one. Whilst we commend pupils for their desire to respect, we question whether a refusal to genuinely engage with claims to knowledge, and therefore to take seriously the people who make them, is respectful at all? Following Barnes, we argue that RE should enable pupils to take differences in knowledge claims seriously. There is a crucial distinction to be made between respecting people because they are fellow human beings and misconstruing respect as uncritical engagement with their claims to knowledge (Barnes 2009).

We are also concerned that the prioritisation of opinion as knowledge which stems from “respect”, constitutes a kind of limited epistemic practice which is akin to early closure (Ashley, 2005). Rather than being prepared for adult life, and indeed enabled for their current lives, such limited epistemic practice may well leave young people epistemically incapacitated when they leave school. The social injustice of this becomes apparent when we consider that not all pupils seem so deprived. In the excerpt below, the second pupil demonstrates a higher level of epistemic literacy than the first—that is, he demonstrates a more advanced understanding of how knowledge works, and can use relevant language more precisely:

Interviewer: Can people’s ideas, beliefs on these questions be wrong?

Pupil one: No. It’s their decision.

Interviewer: You mentioned things like flat earth earlier. If my belief is that the earth is flat, is that right then?

Pupil one: Well, I might say it’s wrong. But in your opinion it’s right, so it’s right.

Pupil two: That’s like taking a scientific approach but not following the scientific part of it.

Pupil one: I don’t think that’s wrong.

Interviewer to pupil two: Say something more about that?

Pupil two: Because if you’re using the scientific approach, you’ve got to say facts about science. You can’t be saying made up stuff and saying it’s fact.

A minority of pupils expressed similarly advanced levels of epistemic literacy. We consider these pupils to be epistemically advantaged in relation to the majority of pupils we spoke to. The first pupil precisely distinguishes between terms, whilst the second presents a relatively sophisticated account (he was 12 years old) of specialist knowledge.

Opinions are what you believe, knowledge is what you've been taught, and facts are what is actually true.

How to throw a normal jab, right, and an uppercut… [T]hat’s the knowledge on boxing… [H]e trained more, and he knew more about boxing and what to do. So his knowledge helped him.

As seems to be the experience of the pupil below, when RE enables the development of epistemic literacy, it is likely to make the big questions harder to answer rather than settling for simple conclusions:

I’ve definitely thought about [big questions] a lot more, since doing them in [RE]… I always used to have my own answer and think that nothing could disprove it… But now I hear lots of evidence, it’s really hard to make a decision now.

### Closing words

Our concern is that respect as a mechanism to avoid personal criticism negates critical engagement. It makes claims to a liberal democracy through recognition of plural objective truths while avoiding the unsettling uncertainty of engaging critically with this plurality. Furthermore, the knowledge claims in the substantive or disciplinary elements of the RE curriculum, in addition to the knowledge claims of the teacher and pupils, simultaneously demand interrogation so as not to control the intellectual culture of the classroom and, by implication, society (see Bordieu 1977) through epistemically unjust avoidance of the dialectic of truths and truth. Indeed, Young’s claim that knowledge is powerful when it enables the search for truth, in its broadest sense (Young, 2015), can never be realised in the epistemic environment that avoids critical engagement with truth.

It will be recalled that one of our research questions asked if the development of epistemic literacy is a viable aim for RE. In this paper, we have attempted to show that at least some pupils are disadvantaged in that they are not enabled to develop their epistemic literacy. Building on Fricker (2007), we contend that such pupils suffer epistemic injustice when they are put at a disadvantage in terms of their abilities to access, recognise and navigate knowledges. We worry that epistemically disadvantaged pupils may be disadvantaged (deprived) in other socially unjust ways, but our data does not speak to this. We recommend this as an area for future research.

We have argued that the prioritisation of epistemic literacy in RE would develop in pupils the wherewithal to handle knowledge claims in their adult and present lives. Following both Ashley (2005) on early closure and Clifford (1877) on the ethics of belief, part of this wherewithal consists in resisting making judgements until knowledge is sufficient. This requires RE teachers to resist asking pupils to conclusively answer (big) questions until an appropriate stage in the development of their epistemic literacy. If we have argued convincingly in this paper, might we have made a case for a single overarching aim of RE expressed, as follows?

RE should develop in learners the lifelong capabilities to develop better knowledge about religion(s)/the religious/the existential.

In suggesting this aim, we are advocating an ethic of RE rather than a particular pedagogy or curriculum. We are familiar enough with at least some existing pedagogical approaches to know that they encompass accounts of what it means to know well and therefore of how to enable learners to develop better knowledge. We hope to have convinced readers that enabling young people to know better matters for young people themselves as well as for society as a whole. Following Clifford (1877) and Haack (2015) epistemic literacy matters for society because of the dangers of a credulous populace. Therefore, we contend that RE has a responsibility to go beyond enabling young people to know better and should expect them to do so.
